# Study of Antidiabetic Properties of Berberis asiatica and Withania somnifera in Streptozotocin-Nicotinamide-Induced Type II Diabetes Mellitus in Wistar Rats

**DOI:** 10.7759/cureus.64379

**Published:** 2024-07-12

**Authors:** Devkumar D Tiwari, Vandana M Thorat, Prathamesh V Pakale, Sarika J Patil

**Affiliations:** 1 Department of Pharmacology, Krishna Vishwa Vidyapeeth (Deemed to be University), Satara, IND

**Keywords:** combination effect, ethanolic plant extract, animal study, diabetes type 2, herbal drugs

## Abstract

Background and aim

Diabetes is a chronic metabolic disorder characterized by elevated blood glucose levels. Although current antidiabetic drugs are highly effective, they are associated with various adverse drug reactions, including life-threatening hypoglycemia, skin rashes, and gastrointestinal intolerance, in addition to being costly. This animal-based experimental study aims to develop a herbal alternative or adjuvant to current antidiabetic drugs using *Berberis asiatica* (*BA*) and *Withania somnifera* (*WS*), which could potentially have fewer adverse drug reactions and reduce the required dose of existing antidiabetic medications.

Material and methods

Seventy-eight adult albino Wistar rats weighing between 150 and 250 g were used for the study. Diabetes mellitus (DM) was induced by intraperitoneal (i.p) injections of streptozotocin (STZ) (65 mg/kg) 15 minutes after nicotinamide (NIC) (110 mg/kg) administration. As the diabetes was confirmed (blood glucose level > 250 mg/dL), rats were divided into 13 different groups mentioned. The standard antidiabetic drugs (metformin [MET] and glimepiride [GLI]) and polyherbal combinations (PHC) (*BA* + *WS*) were administered orally, individually (*WS* and *BA*), and in combination (*BA* + *WS*). Blood samples were collected for biochemical analysis using the tail vein prick method.

The study is based on a total of 13 groups, six rats in each group. Groups 1 and 2 (normal control [NC] and diabetic control [DC]) received distilled water at a dose of 10 mL/kg orally for 28 days. Groups 3-5 (*BA* 250, 500, and 1000) received dried ethanolic root extract of *BA* at a dose of 250, 500, and 1000 mg/kg orally, respectively, for 28 days. Groups 6-8 (*WS* 250, 500, and 1000) received dried ethanolic root extract of *WS* at a dose of 250, 500, and 1000 mg/kg orally, respectively, for 28 days. Groups 9-11 (PHC 250, 500, and 1000) received dried ethanolic root extract of *BA *+ *WS* at a dose of 250, 500, and 1000 mg/kg orally, respectively, for 28 days. Groups 12 and 13 (MET and GLI) received standard drugs MET and GLI at a dose of 250 and 10 mg/kg orally, respectively, for 28 days.

Results

The dried ethanolic root extract of medicinal herbal plants *BA* and *WS* and their combination exhibited significant antidiabetic efficacy. PHC has been shown to have a superior antidiabetic effect than individuals. PHC 500 and 1000 showed blood glucose levels similar to those of the GLI group (P < 0.05). Additionally, PHC 1000 showed blood glucose levels similar to those of the MET group (P < 0.05).

Conclusion

Our results indicate that both *BA *and *WS* possess hypoglycemic activity, and their combination also has a synergistic antidiabetic effect compared to the individual extract. These findings are promising in developing new safe and cost-effective herbal combinations as alternatives or additives to currently used synthetic antidiabetic drugs.

## Introduction

According to the World Health Organization (WHO), diabetes is a chronic metabolic disease characterized by increased levels of blood glucose [1]. Further, diabetes mellitus (DM) manifests as hyperglycemia and, if untreated, can cause damage to various systems, leading to dysfunction or failure of organs, such as the heart, blood vessels, eyes, kidneys, and nerves [2]. Several traditional medicinal plants are known for their antidiabetic properties, including *Terminalia arjuna* [3], *Commiphora mukul* [4], *Phyllanthus emblica* [5], *Azadirachta indica* [6], *Momordica charantia* [7], *Berberis asiatica* (*BA*) [8] and *Withania somnifera* (*WS*) [9]. These herbal plants have a long history of use in various traditional medicines. In Ayurveda, different parts of these plants, such as roots, stems, barks, leaves, seeds, and fruits, are used as phyto-therapeutic agents to treat various health conditions.

*BA* (Indian barberry or Daruharidra [10]) belongs to *Berberidaceae* and is found in Central and Southern Europe, Western Asia, and Northwest Africa [11]. There are about 13 berberis species in the Himalayan region, with heights ranging from 3,000 to 13,000 feet, including *BA*. The root, stem, bark, and leaves of this plant are used to treat ulcers, urethral discharges, ophthalmia, jaundice, fevers, rheumatism, skin disease, dental caries, laxative and tonic, conjunctivitis, and other inflammations of the eyes [12-14]. *WS* (Indian ginseng or winter cherry, also known as ashwagandha [15]) belongs to the family *Solanaceae*. It is a xerophytic plant that grows in Africa, the Mediterranean, Sri Lanka, Pakistan, and India [16]. *WS* acts as an antidote, insecticidal, and larvicidal. The root, stem, leaves, and seeds of *WS* are used for the treatment of asthma, bronchitis, leucoderma, tuberculosis, liver issues, heart disorders, ulcers, swelling, external aches, hemorrhoids, boils, eyesores and edema, and arthritis. This plant has antibacterial, anti-tumor, anticancer, antioxidant, anti-arthritic, anti-inflammatory, immunomodulatory, neurotic regeneration, adaptogenic, nootropic, hypothyroid, herbicidal and pesticidal, abortifacient, astringent, aphrodisiac, emmenagogue, diuretic, narcotic, hypnotic, hepatoprotective, and cardioprotective properties [17].

Both *BA* and *WS* have documented antidiabetic activity in traditional medicine systems, like Ayurveda, the Indian traditional medicinal system “Siddha” [18], and traditional Chinese medicine. Limited research and animal experiments have been conducted to study their antidiabetic activity [19]. If the antidiabetic properties of both herbal plants are confirmed, it could be better herbal medicine and provide effective alternatives or adjuvants to standard treatments for better DM management.

## Materials and methods

Experimental animal

Adult albino Wistar rats weighing 150-250 g were used for the study. The animals were procured from the Central Animal House of Krishna Institute of Medical Sciences, Krishna Vishwa Vidyapeeth (KVV), Satara, India. Animals were kept under standard laboratory conditions, maintaining 12 hours of light/dark cycle at 27-37°C. They had free access to food and water till the end of the study. Approval was taken from the Institutional Ethics Committee (IEC) of KVV (IEC Approval no. 385/2020-2021) and the Institutional Animal Ethics Committee of KVV (Deemed to be University), Reg. No. 255/PO/REBi/S/2000/CPCSEA (IAEC Approval no. IAEC/KIMS/2021/16). All the experiments were done according to guidelines by the Committee for Control and Supervision of Experiments on Animals (CCSEA) in the Central Animal House, KVV. The MET, GLI, *BA*, and *WS* were dissolved in distilled water and given orally. Standard antidiabetic drugs, herbal drugs, and herbal combination dosages were prepared freshly just before the use and given orally from the seventh day daily till the end of the study (35th day). Doses of the drugs and herbal combinations were selected from the previous studies conducted in our laboratory and from the literature.

Acute oral toxicity study

The acute toxicity study was carried out in adult Wistar rats by the “limit dose” method of the Organization for Economic Co-operation and Development (OECD) Guideline No. 240. A test procedure with a starting dose of 2000 mg/kg BW was adopted. The animals were fasted overnight, and the next day, extracts of the plants *BA* and *WS* were administered orally at a dose level of 2000 mg/kg BW. The animals were observed continuously for three hours for general behavioral, neurological, and autonomic profiles, then every 30 minutes for the next three hours, and finally for mortality after 24 hours to 14 days.

Limit test

As per the OECD guidelines, it was ensured that the total dose of the polyherbal combination (PHC) of *BA* + *WS* will not exceed 2000 mg/kg BW, which is considered the upper limit dose to assess acute toxicity. As the first animal was dosed with the upper limit dose and survived, the second animal received the same dose. A total of three animals were dosed with the limited dose, and as no deaths occurred, the three animals of the other sex were tested at the limited dose level. As there was no lethality, the test was terminated.

A total of 78 rats were used in 13 different groups, consisting of six rats in each group for the experiments (Table 1).

**Table 1:** Group distribution This table consists of all 13 groups of distribution of rats with their specific drugs, dosage forms, and route of administration. Each group consists of six rats. NC, normal control; DC, diabetic control; *BA*, *Berberis asiatica*; *WS*, *Withania somnifera*; PHC, polyherbal combination; MET, metformin; GLI, glimepiride

Group no.	Group name	Extract/drugs	Dose and route (orally) mg/kg
1	NC	Distilled water	10 mL/kg
2	DC	Distilled water	10 mL/kg
3	*BA* 250	Dried ethanolic root extract of *BA*	250
4	*BA* 500	Dried ethanolic root extract of *BA*	500
5	*BA* 1000	Dried ethanolic root extract of *BA*	1000
6	*WS* 250	Dried ethanolic root extract of *WS*	250
7	*WS* 500	Dried ethanolic root extract of *WS*	500
8	*WS* 1000	Dried ethanolic root extract of *WS*	1000
9	PHC 250	Dried ethanolic root extract of *BA* + *WS*	125 + 125
10	PHC 500	Dried ethanolic root extract of *BA* + *WS*	250 + 250
11	PHC 1000	Dried ethanolic root extract of *BA* + *WS*	500 + 500
12	MET	Metformin (standard)	250
13	GLI	Glimepiride (standard)	10

Drugs

The dried ethanolic root extract of *BA* and *WS* was procured from Natucare India Pvt. Ltd., Mumbai, India, and Bhagwati Herbal and Healthcare Pvt. Ltd., Vapi, India, in pure powder form. Streptozotocin (STZ) and nicotinamide (NIC) were procured from Sisco Research Laboratories Pvt. Ltd., Mumbai. Metformin (MET) and glimepiride (GLI) were procured from Smruthi Organic Limited, Solapur, India, in pure powder form.

Induction of type 2 DM

Type 2 DM was induced by injecting STZ (65 mg/Kg BW) intraperitoneally (i.p) in physiological saline 15 minutes after NIC (110 mg/Kg BW) administration i.p [19]. The rats were kept and monitored for seven days after the injecting STZ-NIC. Random blood glucose level was examined using the tail vein prick method using a standard portable digital android glucometer (BeatO Curv). The rats who had random blood glucose levels of more than 250 mg/dL on the seventh day were considered diabetic and used for the study [19].

Blood collection

Blood samples were collected by tail vein prick method for estimation of blood glucose level using a portable glucometer.

Statistical analysis

Data analysis was performed using a one-way analysis of variance (ANOVA) followed by a post hoc test. The Tukey Kramer test (post hoc test) was used to identify the statistically significant differences in various groups. Values of blood glucose levels of all the groups are summarized as mean ± SD. All P-value differences were considered statistically significant for P ≤ 0.05. All statistical analyses were performed with SPSS version 11.0 (SPSS Inc., Chicago, IL).

## Results

The study revealed statistically significant changes at all time intervals, specifically on days 0-35, in the study groups DC, *BA* 250, *BA* 500, *BA* 1000, MET, and GLI, with the exception of the NC group. Post hoc analysis showed a statistically significant continuous increase in blood glucose levels in the DC group from days 0 to 35 (P < 0.0001). Concurrently, there was a statistically significant continuous decrease in blood glucose levels in all groups, except NC and DC, from days 7 to 35 (P < 0.0001) (Table 2).

**Table 2:** Effect of BA on blood glucose levels in rats All the values are expressed in mean ± SD. P < 0.05 is considered significant. F and P-values presented in the column are from the repeated ANOVA and Tukey Kramer multiple comparison test. F and P-values presented in the row are from the ordinary ANOVA and Tukey Kramer multiple comparison test. ^a^DC differs significantly from *BA* 250, *BA* 500, *BA* 1000, MET, and GLI groups. ^b^MET differs significantly from DC, *BA* 250, *BA* 500, *BA* 1000, and GLI groups. ^c^GLI differs significantly from DC, *BA* 250, *BA* 500, *BA* 1000, and MET groups. ^1^P < 0.05 ^2^P < 0.01 ^3^P < 0.001 NC, normal control; DC, diabetic control; *BA*, *Berberis asiatica*; MET, metformin; GLI, glimepiride; SD, standard deviation; P, probability value; F, f-statistic

Days/group		0	7th	14th	21st	28th	35th	^Repeated measures ANOVA^		
								F		P
NC		115 ± 12.84	122.17 ± 4.96	116.5 ± 11.98	114 ± 11.47	120.50 ± 2.811	120.17 ± 6.27	0.7779	0.5749	
DC		125.17 ± 12.58	369.33 ± 115.30	405.5 ± 101.05	412.67 ± 114.96	430 ± 108.88	433 ± 94.69	37.39	<0.0001	
*BA* 250		115.5 ± 9.16	456.33 ± 86.25	450.5 ± 85.32^b2^	369.67± 68.79^b2^	297.67±39.92^a3, b3^	255.67±28.75^a3, b3, c3^	74.03	<0.0001	
*BA* 500		112 ± 4.78	517.33 ± 40.29	500.67 ± 38.23^b3, c1^	403.83 ± 24.97^b3, c1^	259.67 ± 11.22^a3, b1^	138.17 ± 11.13^a3^	352.37	<0.0001	
*BA* 1000		110.33 ± 13.56	529.5 ± 30.4	504 ± 22.86^b3, c1^	285.17 ± 5.78^a2^	232.33 ± 4.08^a3^	122.17 ± 3.97^a3^	795.68	<0.0001	
MET		114.33 ± 4.23	407.5 ± 54.48	311.67 ± 47.08	247.67 ± 26.34^a3^	174.17 ± 30.07^a3^	103.17 ± 13.08^a3^	153	<0.0001	
GLI		115.1 ± 18.07	483.33 ± 49.57	390.5 ± 19.94	293.17 ± 10.82^a2^	229 ± 11.66^a3^	121 ± 7.48^a3^	273.83	<0.0001	
Oridnary ANOVA	F	0.9789	28.672	34.542	23.575	27.866	60.150			
	P	0.4541	<0.0001	<0.0001	<0.0001	<0.0001	<0.0001			

**Figure 1:**
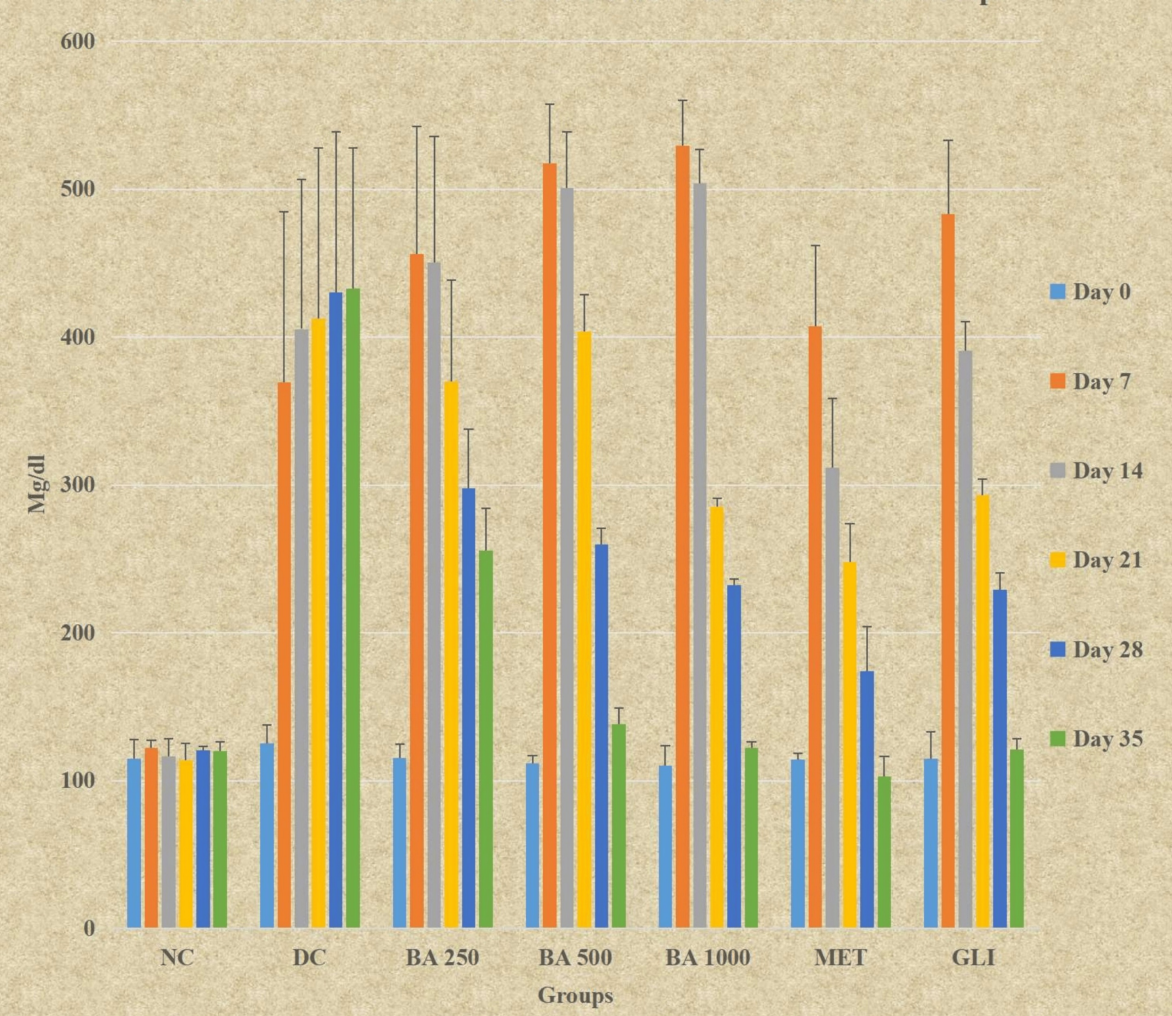
Graphical representation of the effect of Berberis asiatica on blood glucose levels in rats All the data presented is in mean ± SD. SD, standard deviation

Ordinary ANOVA followed by the Tukey Kramer multiple comparison test revealed significant differences in all study groups (NC, DC, *BA* 250, *BA* 500, *BA* 1000, MET, and GLI) at all time intervals (days 7, 14, 21, 28, and 35) except for day 0. The study indicated significantly lower blood glucose levels for *BA* 250 and *BA* 500 on days 28 and 35 compared to DC (P < 0.0001). *BA* 1000 exhibited significantly lower blood glucose levels from days 21 to 35 compared to DC (P < 0.0001). Additionally, *BA* 250 had significantly higher blood glucose levels from days 14 to 35 when compared with MET (P < 0.05) and day 35 compared to GLI (P < 0.0001). *BA* 500 demonstrated significantly higher blood glucose levels from days 14 to 28 compared to MET and on days 14 and 21 compared to GLI (P < 0.05). *BA* 1000 showed significantly higher blood glucose levels on day 14 compared to both MET and GLI (P < 0.05). *BA* 500 had similar blood glucose levels at day 35 compared to MET and on days 28 and 35 compared to GLI (P > 0.05). *BA* 1000 also had similar blood glucose levels when compared to MET and GLI on days 21, 28, and 35 (P > 0.05) (Table 2, Figure 1).

**Figure 2:**
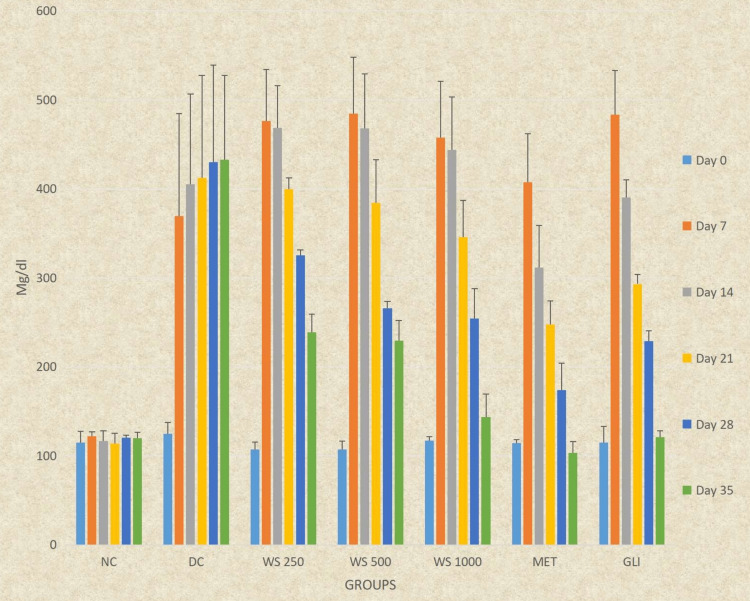
Graphical representation of the effect of Withania somnifera on blood glucose levels in rats All the data presented is in mean ± SD. SD, standard deviation

Repeated measures ANOVA indicated statistically significant differences at all time intervals from days 0 to 35 in all study groups (DC, *WS* 250, *WS* 500, *WS* 1000, MET, and GLI) except for NC. Post hoc analysis by the Tukey Kramer multiple comparison test revealed a statistically significant continuous increase in blood glucose levels in the DC group from days 0 to 35 (P < 0.0001). There was also a statistically significant continuous decrease in blood glucose levels in all groups, except NC and DC, from days 7 to 35 (P < 0.0001) (Table 3, Figure 2).

**Table 3:** Effect of WS on blood glucose levels in rats All the values are expressed in mean ± SD. P < 0.05 is considered significant. F and P-values presented in the column are from the repeated ANOVA and Tukey Kramer multiple comparison test. F and P-values presented in the row are from the ordinary ANOVA and Tukey Kramer multiple comparison test. ^a^DC differs significantly from *WS* 250, *WS* 500, *WS* 1000, MET, and GLI groups. ^b^MET differs significantly from DC, *WS* 250, *WS* 500, *WS* 1000, and GLI groups. ^c^GLI differs significantly from DC, *WS* 250, *WS* 500, *WS* 1000, and MET groups. ^1^P < 0.05 ^2^P < 0.01 ^3^P < 0.001 NC, normal control; DC, diabetic control; *WS*, *Withania somnifera*; MET, metformin; GLI, glimepiride; SD, standard deviation; P, probability value; F, f-statistic

Days/group	0	7th	14th	21st	28th	35th	^®^F	^®^P
NC	115 ± 12.84	122.17 ± 4.96	116.5 ± 11.98	114 ± 11.47	120.50 ± 2.811	120.17 ± 6.27	0.7779	0.5749
DC	125.17 ± 12.58	369.3± 115.30	405.5 ± 101.05	412.67 ± 114.96	430 ± 108.88	433 ± 94.69	37.39	<0.0001
*WS *250	107.33 ± 7.99	476.5 ± 57.37	468.83 ± 47.21^b3^	399.83 ± 12.77^b3,c1^	325.50 ± 6.22^a2, b3, c1^	239 ±20.49^a3, b3, c3^	168.63	<0.0001
*WS *500	107.17 ± 9.432	484.83 ± 63.18	468.17 ± 60.88^b3^	384.33 ± 48.58^b3^	266 ± 7.59^a3, b1^	229.67 ± 22.30^a3, b3, c3^	155.51	<0.0001
*WS* 1000	117.17 ± 4.22	457.5 ± 63.49	444 ± 59.34^b2^	345.67± 41.25^b1^	254.50 ± 33.45^a3^	143.67 ± 25.88^a3^	149.25	<0.0001
MET	114.33 ± 4.23	407.5 ± 54.48	311.67 ± 47.08	247.67 ± 26.34	174.17 ± 30.07	103.17 ± 13.08	153	<0.0001
GLI	115.1± 18.07	483.33 ± 49.57	390.5 ± 19.94	293.17 ± 10.82	229 ± 11.66	121 ± 7.48	273.83	<0.0001
F	1.894	23.636	29.402	25.851	30.352	52.948	One-way ANOVA ®-repeated ANOVA	
P	0.1095	<0.0001	<0.0001	<0.0001	<0.0001	<0.0001		

Ordinary ANOVA followed by post hoc analysis revealed significant differences in all study groups (NC, DC, *WS* 250, *WS* 500, *WS* 1000, MET, and GLI) at all time intervals (days 7 to 35) except for day 0 (P < 0.0001). *WS* 250, *WS* 500, and *WS* 1000 showed significantly lower blood glucose levels on days 28 and 35 compared to DC (P < 0.0001). *WS* 250 and *WS* 500 had significantly higher blood glucose levels from days 14 to 35, and *WS* 1000 had significantly higher levels on days 14 and 21 compared to MET (P < 0.0001). Compared to GLI, *WS* 250 had significantly higher blood glucose levels from days 21 to 35 and *WS* 500 on day 35 (P < 0.05). *WS* 500 had similar blood glucose levels at all time intervals (days 0 to 28) compared to GLI (P > 0.05) except for day 35. *WS* 1000 had similar blood glucose levels at days 28 and 35 compared to MET (P > 0.05) and at all intervals (days 0 to 35) compared to GLI (P > 0.05) (Table 3, Figure 2).

**Figure 3:**
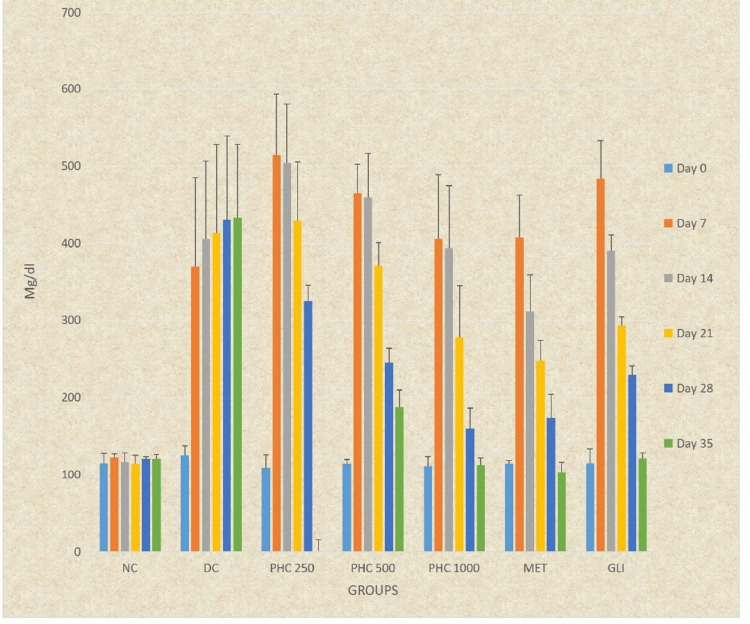
Graphical representation of the effect of PHC on blood glucose level in rats All the data presented is in mean ± SD. PHC, polyherbal combination; SD, standard deviation

Repeated measures ANOVA revealed statistically significant differences at all times from days 0 to 35 in the study groups (DC, PHC 250, PHC 500, PHC 1000, MET, and GLI) except for NC. Post hoc analysis by the Tukey Kramer multiple comparison test indicated a statistically significant continuous increase in blood glucose levels in the DC group from days 0 to 35 (P < 0.0001) and a statistically significant continuous decrease in blood glucose levels in all groups, except NC and DC, from days 7 to 35 (P < 0.0001) (Table 4, Figure 3).

**Table 4:** Effect of PHC on blood glucose levels in rats All the values are expressed in mean ± SD. P < 0.05 is considered significant. F and P-values presented in the column are from the repeated ANOVA and Tukey Kramer multiple comparison test. F and P-values presented in the row are from the ordinary ANOVA and Tukey Kramer multiple comparison test. ^a^DC differs significantly from PHC 250, PHC 500, PHC 1000, MET, and GLI groups. ^b^MET differs significantly from DC, PHC 250, PHC 500, PHC 1000, and GLI groups. ^c^GLI differs significantly from DC, PHC 250, PHC 500, PHC 1000, and MET groups. ^1^P < 0.05 ^2^P < 0.01 ^3^P < 0.001 NC, normal control; DC, diabetic control; PHC, polyherbal combination; MET, metformin; GLI, glimepiride; SD, standard deviation; P, probability value; F, f-statistic

Day/group	0	7th	14th	21st	28th	35th	^®^F	^®^P
NC	115 ± 12.84	122.17 ± 4.96	116.5 ± 11.98	114 ± 11.47	120.50 ± 2.811	120.17 ± 6.27	0.7779	0.5749
DC	125.17 ± 12.58	369.33 ± 115.30	405.5 ± 101.05	412.67 ± 114.96	430 ± 108.88	433 ± 94.69	37.39	<0.0001
PHC 250	108.83± 17.12	513.50 ± 79.56^a1 ^	504 ± 76.26^b3^	429.17± 75.83^b3, c2^	324.83 ± 20.28^a2, b3, c1^	298 ± 15.17^a3, b3, c3^	89.15	<0.0001
PHC 500	114.5 ± 5.61	464.33 ± 37.98	458.83 ± 57.26^b2^	370.50± 30.05^b1^	245.17 ±18.18^a3, ^	187.50 ± 21.92^a3, b2^	223.22	<0.0001
PHC 1000	111.5 ± 12.28	405.33 ± 83.45	393.33 ± 81.16	277.67 ± 66.76^a2^	159.67 ± 26.31^a3^	112.67 ± 8.94^a3, ^	65.11	<0.0001
MET	114.33 ± 4.23	407.5 ± 54.48	311.67 ± 47.08	247.67± 26.34	174.17 ± 30.07	103.17 ± 13.08	153	<0.0001
GLI	115.1± 18.07	483.33 ± 49.57	390.5 ± 19.94	293.17 ± 10.82	229 ± 11.66	121 ± 7.48	273.83	<0.0001
F	0.9455	21.334	23.388	19.93	33.574	65.360	One-way ANOVA ®-repeated ANOVA	
P	0.4756	<0.0001	<0.0001	<0.0001	<0.0001	<0.0001		

Ordinary ANOVA followed by post hoc analysis showed significant differences in all study groups (NC, DC, PHC 250, PHC 500, PHC 1000, MET, and GLI) at all time intervals (days 7 to 35) except for day 0 (P < 0.0001). PHC 250 and PHC 500 had significantly lower blood glucose levels on days 28 and 35 and PHC 1000 from days 21 to 35 compared to DC (P < 0.0001). PHC 250 had significantly higher blood glucose levels from days 14 to 35 and PHC 500 from days 14 to 35 compared to MET (P < 0.0001), except for day 28. PHC 250 had significantly higher blood glucose levels from days 21 to 35 compared to GLI (P < 0.05). PHC 500 and PHC 1000 had similar blood glucose levels at all time intervals (days 0 to 35) compared to GLI (P > 0.05). PHC 1000 also had similar blood glucose levels at all time intervals compared to MET (P > 0.05) (Table 4, Figure 3).

## Discussion

The current study evaluated the antidiabetic properties of *BA* and *WS* in an STZ-NIC-induced type II DM model in Wistar rats. The results revealed significant antidiabetic effects of both herbal extracts and their PHC, as evidenced by the reduction in blood glucose levels across multiple time intervals. *BA* and *WS* are traditionally used medicines for beating ulcers, jaundice, fever, etc, which was reported in a study by Srivastava et al. (2004) [13]. Animal and pilot studies conducted by Singh and Jain (2010), Khan et al. (2017), and Thakur et al. (2015) suggest the antidiabetic effect of BS [8,20] and *WS* [21].

Glycemic control

Berberis asiatica

Berberine has been shown to lower blood glucose levels, potentially raise insulin secretion, decrease body weight and lipid levels, increase glucagon-like peptide-1 (GLP-1) levels, and possess significant DPP-IV inhibitory activity [22,23]. *BA*, commonly known as Indian barberry, is rich in berberine, an alkaloid with known antidiabetic properties. Berberine has been shown to improve insulin sensitivity, enhance glucose uptake in peripheral tissues, and reduce hepatic gluconeogenesis (Yin et al., 2008; Kong et al., 2009) [24,25]. The study demonstrated that *BA* at various dosages (250, 500, and 1000 mg/kg) resulted in significant reductions in blood glucose levels in diabetic rats compared to the diabetic control (DC) group. Post hoc analysis indicated a continuous and statistically significant decrease in blood glucose levels from days 7 to 35 for all *BA* treatment groups (P < 0.0001), in contrast to the DC group, which exhibited a significant increase in blood glucose levels over the same period (P < 0.0001).

Withania somnifera

*WS*, commonly known as Ashwagandha, has been traditionally used in Ayurvedic medicine for its adaptogenic and therapeutic properties. Previous studies have demonstrated its role in modulating insulin secretion and improving glucose metabolism (Mishra et al., 2000; Verma and Singh, 2012) [26,27]. *WS* inhibits carbohydrate breakdown and α-glucosidase enzyme and stimulates pancreatic β-cell, glucose transporters, and insulin receptors, which will increase the insulin secretion and transportation of glucose into the cell and decrease the insulin resistance, respectively [28]. Our findings are consistent with these reports, as *WS* treatment also led to significant reductions in blood glucose levels at all tested dosages (250, 500, and 1000 mg/kg) compared to the DC group (P < 0.0001). The continuous decrease from days 7 to 35 for *WS* groups highlights its potential in managing hyperglycemia in diabetic rats.

Polyherbal combination

As we know, there is a presence of berberine in *BA* and flavonoids in *WS*. Berberine and flavonoid both are potential antidiabetic constituents reported by Belwel et al. (2020) and Udayakumar et al. (2009) [29,30]. The combined treatment of *BA* and *WS* showed a synergistic effect, leading to a more pronounced reduction in blood glucose levels compared to individual treatments. This suggests potential complementary mechanisms of action, where *BA* enhances insulin sensitivity and *WS* promotes insulin secretion, collectively contributing to better glycemic control.

Dose-dependent efficacy

The highest dose of *BA* (1000 mg/kg) showed the most pronounced reduction in blood glucose levels, achieving levels comparable to those in the MET and GLI groups from days 21 to 35 (P > 0.05). These findings suggest a dose-dependent effect of *BA*, with higher doses offering greater glycemic control. The *WS* 1000 group showed similar efficacy to the MET and GLI groups from days 28 to 35 (P > 0.05), suggesting that high-dose of *WS* can achieve comparable glycemic control to standard antidiabetic drugs. *WS* 250 and *WS* 500 groups had significantly higher blood glucose levels compared to the MET and GLI groups at earlier time points, but *WS* 500 showed similar levels to the GLI group at all intervals except on day 35. PHC 250 and PHC 500 groups demonstrated significantly lower blood glucose levels on days 28 and 35 compared to the DC groups (P < 0.0001). PHC 250 and PHC 500 groups also demonstrated a significantly higher blood glucose level as compared to the MET group on days 14, 21, 28, and 35 (P < 0.0001). PHC 250 also had higher blood glucose levels as compared to the GLI group on days 21, 28, and 35 (P < 0.05). The PHC 1000 group showed a significantly lower blood glucose level as compared to the DC group from days 21 to 35 (P < 0.0001). PHC 500 and PHC 1000 groups showed blood glucose levels similar to those of the GLI group at all testing time intervals (P > 0.05). PHC 1000 showed blood glucose levels similar to those of the MET group at all testing time intervals (P > 0.05).

Comparison with standard treatments

While *BA* 250 and *BA* 500 groups showed significantly higher blood glucose levels compared to the MET group at earlier time points (days 14 to 28 for *BA* 500 and days 14 to 35 for *BA* 250), *BA* 500 demonstrated statistically similar levels to the MET group by day 35. *BA* 1000 exhibited comparable efficacy to both MET and GLI groups by day 21, indicating that high-dose *BA* might be as effective as conventional antidiabetic medications. *WS* 1000 demonstrated efficacy comparable to MET from day 28 and to GLI from day 21, highlighting its potential as an effective antidiabetic treatment at higher doses. *WS* 250 and *WS* 500, while effective, did not achieve the same level of glycemic control as *WS* 1000. PHC 1000 demonstrated efficacy comparable to MET and GLI, whereas PHC 500 is only comparable to GLI at all the testing time intervals, pointing out that higher dosage can be as effective as conventional antidiabetic medications.

Mechanistic insights

The observed antidiabetic effects of *BA* and *WS* likely involve multiple pathways that could be attributed to their active components and their mechanisms of action. Berberine, the active compound in *BA*, is known to activate AMP-activated protein kinase (AMPK), which enhances insulin sensitivity and promotes glucose uptake in peripheral tissues (Yin et al., 2008; Kong et al., 2009; Lee et al., 2006) [24,25,31]. *WS*, or Ashwagandha, is known for its adaptogenic properties and has been shown to modulate oxidative stress and inflammation, key factors in the pathogenesis of diabetes (Mishra et al., 2000; Verma and Singh, 2012; Choudhary et al., 2017) [26,27,32].

Limitation

Only single species, i.e., albino Wistar rats, were used. In our setup, we were unable to quantify the active principle present in the extract. The efficacy of the extract as an antidiabetic agent can be further evaluated using other suitable animal models in different species and variants. The efficacy of the extract could be evaluated in human studies.

## Conclusions

In conclusion, the study provides compelling evidence for the antidiabetic properties of BA and WS, both individually and in combination. These findings suggest that these herbal extracts could be developed as potential and promising therapeutic agents. It will be a step forward toward the development of new safe and herbal alternatives to presently used synthetic antidiabetic drugs without their side effects and cost-efficiently, which will be useful for patients suffering from diabetes. Further research, including clinical trials, is warranted to confirm these effects in humans and elucidate the precise molecular mechanisms involved.

## References

[1] (2024). Diabetes. https://www.who.int/health-topics/diabetes#tab=tab_1.

[2] American Diabetes Association (2010). Diagnosis and classification of diabetes mellitus. Diabetes Care.

[3] Mohanty IR, Kumar CS, Borde M (2021). Antidiabetic activity of Commiphora mukul and Phyllanthus emblica and computational analysis for the identification of active principles with dipeptidyl peptidase IV inhibitory activity. Indian J Pharmacol.

[4] Mohanty IR, Borde M, Kumar C S, Maheshwari U (2019). Dipeptidyl peptidase IV Inhibitory activity of Terminalia arjuna attributes to its cardioprotective effects in experimental diabetes: In silico, in vitro and in vivo analyses. Phytomedicine.

[5] Huang SM, Lin CH, Chang WF, Shih CC (2023). Antidiabetic and antihyperlipidemic activities of Phyllanthus emblica L. extract in vitro and the regulation of Akt phosphorylation, gluconeogenesis, and peroxisome proliferator-activated receptor α in streptozotocin-induced diabetic mice. Food Nutr Res.

[6] Ezeigwe OC, Ezeonu FC, Igwilo IO (2020). Antidiabetic property and antioxidant potentials of ethanol extract of Azadirachta indica leaf in streptozotocin-induced diabetic rats. Bioscientist J.

[7] Xu X, Shan B, Liao CH, Xie JH, Wen PW, Shi JY (2015). Anti-diabetic properties of Momordica charantia L. polysaccharide in alloxan-induced diabetic mice. Int J Biol Macromol.

[8] Singh P, Jain SK, Alok S (2010). Antidiabetic activity of Berberis asiatica (DC) roots. Int J Pharm Sci Res.

[9] Durg S, Bavage S, Shivaram SB (2020). Withania somnifera (Indian ginseng) in diabetes mellitus: a systematic review and meta-analysis of scientific evidence from experimental research to clinical application. Phytother Res.

[10] Sher H, Jan H, Ur-Rahman I (2021). Berberis aristata DC. Berberis asiatica Roxb. ex DC. Berberis chitria Buch.-Ham. ex D. Don Berberis glaucocarpa Stapf Berberis lycium Royle Berberis orthobotrys Bien. ex Aitch. ssp. orthobotrys Berberis vulgaris L. BERBERIDACEAE. Ethnobotany of the Himalayas.

[11] Salehi B, Selamoglu Z, Sener B (2019). Berberis plants-drifting from farm to food applications, phytotherapy, and phytopharmacology. Foods.

[12] (2024). Herb latin name: Berberis asiatica. https://www.naturalmedicinalherbs.net/herbs/b/berberis-asiatica.php.

[13] Srivastava SK, Singh Rawat AK, Mehrotra S (2004). Pharmacognostic evaluation of the root of Berberis asiatica. Pharm Biol.

[14] (2024). Berberis asiatica - asian barberry. https://www.flowersofindia.net/catalog/slides/Asian%20Barberry.html.

[15] Rathi V, Dhingra AK, Chopra B (2021). Withania somnifera. Naturally Occurring Chemicals Against Alzheimer’s Disease.

[16] Shirin K, Imad S, Shafiq S, Fatima K (2010). Determination of major and trace elements in the indigenous medicinal plant Withania somnifera and their possible correlation with therapeutic activity. J Saudi Chem Soc.

[17] Saleem S, Muhammad G, Hussain MA, Altaf M, Bukhari SN (2020). Withania somnifera L.: Insights into the phytochemical profile, therapeutic potential, clinical trials, and future prospective. Iran J Basic Med Sci.

[18] Sharma S, Chaitanya MVNL, Sharma S (2024). The medicinal plant Berberis aristata and its endophytes for pharmacological applications: current research and future challenges. J Appl Biol Biotech.

[19] Rani R, Dahiya S, Dhingra D, Dilbaghi N, Kaushik A, Kim KH, Kumar S (2019). Antidiabetic activity enhancement in streptozotocin + nicotinamide-induced diabetic rats through combinational polymeric nanoformulation. Int J Nanomedicine.

[20] Khan MM, Rastogi C, Gupta S, Paswan SK, Verma P, Jawaid T, Rao CV (2017). Protective effect of Berberis asiatica root on biochemical and histopathological changes in streptozotocin-induced diabetic Wistar rats. Int J Basic Clin Pharmacol.

[21] K Thakur A, Dey A, S Chatterjee S, Kumar V (2015). Reverse ayurvedic pharmacology of ashwagandha as an adaptogenic anti-diabetic plant: a pilot study. Curr Tradit Med.

[22] Utami AR, Maksum IP, Deawati Y (2023). Berberine and its study as an antidiabetic compound. Biology (Basel).

[23] Mohanty IR, Kumar S, Suman R (2017). Dipeptidyl peptidase IV inhibitory activity of berberine and mangiferin: an in silico approach. Int J Clin Endocrinol Metab.

[24] Yin J, Gao Z, Liu D, Liu Z, Ye J (2008). Berberine improves glucose metabolism through induction of glycolysis. Am J Physiol Endocrinol Metab.

[25] Kong WJ, Zhang H, Song DQ (2009). Berberine reduces insulin resistance through protein kinase C-dependent up-regulation of insulin receptor expression. Metabolism.

[26] Mishra LC, Singh BB, Dagenais S (2000). Scientific basis for the therapeutic use of Withania somnifera (ashwagandha): a review. Altern Med Rev.

[27] Verma S, Singh SP (2008). Current and future status of herbal medicines. Vet World.

[28] Kumar V, Dey A, Chatterjee SS (2017). Phytopharmacology of ashwagandha as an anti-diabetic herb. Science of Ashwagandha: Preventive and Therapeutic Potentials.

[29] Belwal T, Bisht A, Devkota HP (2020). Phytopharmacology and clinical updates of Berberis species against diabetes and other metabolic diseases. Front Pharmacol.

[30] Udayakumar R, Kasthurirengan S, Mariashibu TS (2009). Hypoglycaemic and hypolipidaemic effects of Withania somnifera root and leaf extracts on alloxan-induced diabetic rats. Int J Mol Sci.

[31] Lee YS, Kim WS, Kim KH (2006). Berberine, a natural plant product, activates AMP-activated protein kinase with beneficial metabolic effects in diabetic and insulin-resistant states. Diabetes.

[32] Choudhary D, Bhattacharyya S, Bose S (2017). Efficacy and safety of ashwagandha (Withania somnifera (L.) Dunal) root extract in improving memory and cognitive functions. J Diet Suppl.

